# Contracting ‘person-centred’ working by results: street-level managers and frontline experiences in an outcomes-based contract

**DOI:** 10.1080/14719037.2024.2342398

**Published:** 2024-04-18

**Authors:** Eleanor Carter, Franziska Rosenbach, Fernando Domingos, Felix-Anselm van Lier

**Affiliations:** a Blavatnik School of Government, University of Oxford, Oxford, UK; b Sao Paulo School of Business Administration, FGV-EAESP, Sao Paulo, Brazil

**Keywords:** Outcomes-based contracting, governance modes, street-level management, social impact bond, personalization

## Abstract

Outcomes-based contracting (OBC) has been heralded as a mechanism for improving the efficiency and effectiveness of social programmes yet has persistently failed to deliver meaningful support for people experiencing social disadvantage. This mixed-method study evaluates the contractual shift of a British support service for adults with multiple, complex needs from bilateral fee-for-service arrangements to an outcome contract in the form of a ‘social impact bond’. Our findings add much-needed empirical evidence on the implications of OBC for personalization and co-production of public service delivery. In contrast to prior payment-by-results experiments we find evidence of enhanced person-centredness and asset-based practice.

## Introduction

In the pursuit of more efficient, effective and innovative welfare services, governments have pioneered the use of a range of contractual mechanisms to shape the interactions with delivery agencies – what we refer to as ‘street level organisations’ (SLOs) – from the private and social sectors. Although historically ‘street-level bureaucracies’ constitute the government (i.e. public-sector) agencies that directly deliver programmes to citizens, nowadays (following successive waves of outsourcing) non-government organizations often perform this role. Contemporary scholarship tends to talk of ‘SLOs’ since street-level work may be enacted by a variety of organizational forms (Brodkin 2015). Importantly, the work of SLOs is structured through contracts and the connections between alternative contracting and governance arrangements, managerial practice and the work of street-level bureaucrats (SLBs) call for greater scholarly attention (Chang and Brewer 2022). Outcomes-based contracting (OBC and its synonyms such as payment-by-results) is a contractual innovation in which payment to SLOs is conditioned on the successful achievement of pre-agreed, measurable goals or programme outcomes (Lagarde et al. 2013). Initially applied to activating ‘welfare-to-work’ programmes, OBC is now used across a range of policy domains (Carter and Ball 2023; Lazzarini 2022), and in this study, is applied to housing-related support services that are intended to prevent disadvantaged adults from becoming homeless.

Amongst proponents, OBC rectifies incentivization issues seen in more conventional input receipt-based or fee-for-activity contracting arrangements between governments and SLOs by completing the contract and paying for providers’ performance against well-designed outcome metrics (Lazzarini 2022). By removing the specification of activities and services in social programmes, as when OBC is combined with a non-prescriptive ‘black box’ delivery model, the expectation is that non-governmental SLOs have the freedom to innovate and provide effective, tailored support to each individual service user. The justification for OBC moves with the grain of Lipsky’s foundational work: if it is the decisions and practices of SLB as they work (under pressure) that ‘effectively *become* the public policies they carry out’ (Lipsky 1980, xii original emphasis) then the organizational imperatives of street-level calculus should be oriented to ‘social outcomes’ and ‘impact’ (Carter et al. 2018).

Yet the promise of meaningful, personalized, services under payment-by-results arrangements has not always been realized and there are known difficulties for marketized arrangements to enhance innovation, prevent poor quality services and increase efficiency and effectiveness (Carter and Whitworth 2015; Larsen and Wright 2014; McGann 2022). A novel permutation of OBCs is a contracting innovation known as a social impact bond and in contrast to mainstream payment-by-results arrangements, these impact-focused partnerships intentionally involve social-sector delivery organizations and integrate up-front working capital from socially motivated third-party investors. Social-sector providers are shielded from the high-powered incentive contract and instead (some of) the financial risk of underperformance is transferred to a third-party social investor (Carter et al. 2018; FitzGerald et al. 2021). Despite considerable policy hype, it is not clear whether this new mode of OBC can better respond to the ambition for personalized services in complex implementation environments (Wilson et al. 2020) and this is the analytical focus of the study.

One of the challenges in investigating the implication of OBC for frontline practice is that such reforms have typically been introduced alongside other large-scale shifts in market stewardship and service management. This study provides a rare opportunity to investigate the introduction of black box OBC using novel multi-layered (spanning contract and contract management; SLO managers (Street Level Managers, SLMs) and frontline staff (Street Level Workers, SLWs)) and longitudinal data collected with the same eight SLOs as they simultaneously undergo a major contractual reform. We present an analysis of a British support service for adults with multiple, complex disadvantages which has historically been contracted by Kirklees Council, a local authority in northern England, from not-for-profit service providers using bilateral fee-for-service arrangements. Our research data spans a period pre- and post- the introduction of an impact bond across this network of SLOs.

There are tensions in the implications of OBC for street-level practice, particularly in relation to personalized service efforts. Rhetorically, black box OBCs not only accept discretion as an inherent feature of implementation but actively seek to expand discretion at the SLO level, to propagate more personalized and effective support. However, OBC, when framed as an extreme form of New Public Management, brings pressures to hit targets and minimize costs, leading to more *standardized* (rather than personalized) services (Fuertes and Lindsay 2016). It is these tensions between low-cost standardization and effective, substantive personalization that we investigate, and which ground our two central contributions. Empirically, we offer a novel longitudinal analysis of the implementation of a new outcomes-based contracting regime. The longitudinal design is critical for capturing the interplay between contractual shifts and implications for personalization and co-production over time. Our mixed-method design traces changing personalization practice and reflections amongst both SLWs and SLMs as they transition to a black box impact bond contracting arrangement. We make a theoretical contribution by refining the construct of ‘financialised discretion’ and connecting this with street-level management (e.g. Gassner and Gofen 2018), underscoring the importance of governance, resources and management conditions in mediating frontline workers’ personalization practices.

## Literature review

### Street-level management conditions and personalisation practices under outcome-based contracts

A lively literature on street-level bureaucracy and SLOs explores the connections between the governance of public services and the substantive aspects of their provision, content and emphasis at the frontline. SLB theorists such as Brodkin draw attention to the ways that governance and managerial forces shape ‘how street-level organizations and frontline staff deploy their policy discretion “in patterned ways”’ (Brodkin, 2012, 943 in O’Sullivan, McGann, and Considine 2021). Contextual factors frame and shape frontline workers room for discretion by framing and constraining options (Caswell and Larsen 2017). Under contractualized arrangements, government purchasers direct the choices of SLBs at a distance by applying performance incentives and sanctions, expressing and regulating minimum service standards and monitoring delivery (O’Sullivan, McGann, and Considine 2021).

‘Frontline workers react to changing policies and institutional settings’ (Caswell and Larsen 2017, 170). In a series of comparative longitudinal and cross-country investigations, Considine and colleagues explore the changing patterns of frontline decision-making and time use chronicling the rise of more ‘enterprising’ forms of governance with shifts from bureaucratic (or procedural), to managerial (or corporate), market, and network (Considine and Lewis 2003; Considine, Lewis, and Sullivan 2011). The shift towards marketized governance modes and particularly the rise of ‘payment-by-results’ and OBC has been linked to the aspiration for more effective, ‘personalised’ services’ (Considine et al. 2020; Lazzarini 2022; Sainsbury 2017). When OBCs are linked with an intentionally under-specified ‘black box’ delivery model, SLOs are expected to have increased flexibility for tailored, person-led services (Carter et al. 2018; Fox et al. 2022; Lane et al. 2013). The term ‘black box’ can be understood as concealing the early-middle portion of a programme logic model: inputs; activities; and near-term outputs are inside the black box and therefore under the discretion of the SLO.^1^

Affording SLOs great flexibility over implementation whilst enshrining accountability for outcomes and hence simultaneously transferring performance risk to these delivery organizations (since their income is heavily dependent on results) has been described as *financialised discretion* (Considine et al. 2020). It is this financialised discretion that is expected to drive providers to focus on results, develop new practices and pursue innovative, tailored approaches (Finn 2012; Wooldridge, Stanworth, and Ronicle 2019). This flexibility is expected to unlock personalization, where personalization is understood as the tailoring of services to account for individual clients’ needs and aspirations, allowing for some degree of user participation and co-production (Needham 2011b).

Given the heterogeneous personalization effects found under a range of contracting reforms, it is helpful to consider the distinction between two types of personalization (Toerien et al. 2013):

- **Procedural personalization** describes the process underpinning SLW–participant interaction, i.e. the ‘how’ of service provision. It embraces ‘rule discretion’ relating to the autonomy in the frequency and manner of support provision (Rice 2017).
- **Substantive personalization** describes the extent to which the substance of the service is tailored to individual need, i.e. the ‘what’ of service provision. It embraces ‘task discretion’, offering relative autonomy over the content of service to SLWs. It is stipulated that the availability of flanking social services increases the potential for service personalization in street-level practice (Rice 2017).

The financialised discretion of black box OBC may not always or predictably unlock personalization: ‘what we find inside the black box is not always more flexible and adaptive than what is written on the lid’ (O’Sullivan, McGann, and Considine 2021, 50). Although it is not always clear whether the discretion that sits at the heart of the espoused OBC mechanism is expected to be sited at the organizational level or by cascading incentives and flexibilities to SLWs, prior research identifies three key routes by which the espoused personalization of OBC may be undermined. Firstly, studies caution that increasing SLO’s financial accountability for results and exposure to economic risk may intensify processes of standardization and reduce service quality (Brodkin 2011; Fuertes and Lindsay 2016; Larsen and Wright 2014). Under OBCs, providers may respond to financialised performance pressures by retreating to bland, all-purpose, low-cost interventions (O’Sullivan, McGann, and Considine 2021). McGann (2023) finds a reduction in qualifications and professional experience amongst SLW following marketization reforms. In Australian research, employment pathway plans that were expected to be highly tailored are [in fact], pre-filled templates with little agency for participants to shape the contents of the plans (O’Sullivan, McGann, and Considine 2021).

Secondly, extreme forms of OBC raise questions around the diversity of SLOs in terms of sector (the balance of private/non-profit) but also in terms of size, locality and orientation to supporting particular groups of service participants (Shutes and Taylor 2014). A large-scale review of payment-by-results models indicates that social-sector providers may struggle to absorb the risks inherent in scheme design and that good existing provision may be lost (Mason et al. 2015). With continued application of OBC, there is concern that provider markets may become dominated by a limited number and high concentration of non-specialist providers (as seen in UK welfare-to-work and probation services) and this counters arguments for OBC increasing market supply and driving innovation (Mason et al. 2015).

Thirdly, concerns around financialised discretion often centre on the gaming practices of creaming and parking: provider behaviour that prioritizes programme participants who are seen as easier (and cheaper) to support and who will release outcome payments whilst deliberately neglecting those who face more substantial or compound barriers (Carter and Whitworth 2015; Koning and Heinrich 2013; Scarano 2023). Clearly such practices cut against substantive personalization since those who are understood as harder to help may not be receiving even a basic package of support, let alone a person-centred set of flanking and wraparound support services (O’Sullivan, McGann, and Considine 2021; Rees, Whitworth, and Carter 2014).

Our empirical case offers a divergent and potentially pioneering outcomes-based contracting arrangement when read alongside prior studies since impact bonds – at least rhetorically – have emerged as an antidote to the concerns around the absence of innovation, neglect of social-sector service providers and poor specification of outcome measures seen in large-scale OBC arrangements (c.f. Carter 2019). Impact Bonds tend to be smaller scale OBC innovations that open up opportunities for socially motivated service providers (Government Outcomes Lab, n.d.; Roberts 2013). In the archetypal impact bond arrangement, the key appeal for voluntary sector SLOs is that they are *not* required to bear the financial risk of a payment-by-results contract (Carter 2020). The capital required to deliver services is provided by independent, socially motivated investors. This social investment capital is expected to shoulder the risk of non-delivery of outcomes in pursuit of ‘blended’ returns: financial *and* social (Carter 2020; Economy, Carter, and Airoldi 2022).

It is this specific contextual setting and intertwined set of social and financial imperatives that motivates our study. What are the implications for personalization when a black box OBC reform is developed at a local level, with the involvement of longstanding social-sector providers and with pre-financing made available via an explicit social investment fund manager? Our guiding research question asks *what are the implications for managerial emphasis and SLW orientation to personalization following the introduction of a* *major outcomes-based contracting reform in a* *housing support service in Kirklees?*

## Empirical setting

This study provides a rare opportunity to investigate the introduction of a black box outcomes contract across a stable set of SLOs with data collection both before and after the contracting reform. We draw from unique data on the Kirklees Better Outcomes Partnership (KBOP), a social impact bond commissioned under the Life Chances Fund in the United Kingdom (Rosenbach et al. 2023). This is a service for adults experiencing multiple, complex needs and who may, without support, be at increased risk of homelessness. The criteria for accessing the service are intentionally flexible and those engaging may be people with a history of repeat homelessness, offenders, people with mental health problems, learning disabilities, those that abuse substances, those at risk of domestic abuse, care leavers or young parents and refugees.

**Figure 1.**
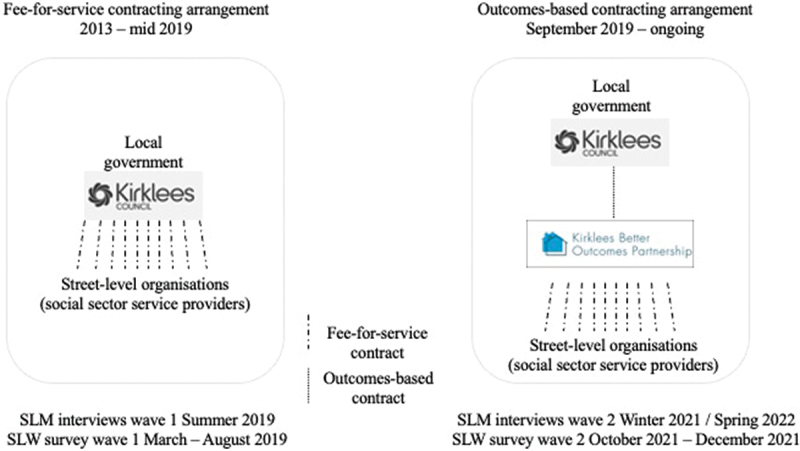
Contractual arrangements for support services in Kirklees. The left-hand pane shows the legacy, bilateral fee-for-service contracting arrangement and the right-hand pane shows the configuration of contracts in the social impact bond.

Participation in the service is voluntary and although the service may support transitions to employment the service is not part of mainstream employability services. Prior to the impact bond, nine voluntary sector providers (SLOs) had delivered services under direct fee-for-service contracts with Kirklees Council (left-hand pane in Figure 1).

In 2019, the impact bond is launched and Kirklees Council (a local government commissioner) shifts from contracting for ‘services’ and instead commits to pay for a series of individual participant-level outcome measures such as maintaining suitable, safe accommodation for 3, 6, 12 or 18 months; maintaining stability and wellbeing; entering and sustaining employment. Table 1 summarizes the distinctions between the fee-for-service contracts and the impact bond arrangements. The outcomes contract is held between the Council and a newly constituted special-purpose vehicle (The ‘KBOP’ SPV, described in the right-hand pane of Figure 1). As a broker organization, the SPV is responsible for holding service delivery contracts with the SLOs^2^ and managing performance. In our setting, these are the same social sector SLOs that have historically been delivering under direct fee-for-service contracts with the council. Interviewees (SLMs) and all survey respondents (SLWs) were working for these SLOs.

**Table 1.** Differences and similarities between legacy fee-for-service contracting and outcomes-based contracting arrangement in Kirklees.

Governance characteristics	Legacy fee-for-service contracts (pre- mid-2019)	Impact bond contracting arrangement (September 2019 onwards)
Commissioning agency	Kirklees Council	
Target cohort	Adults experiencing disadvantage and who may, without support, be at increased risk of homelessness	
Delivered by	9 not-for-profit organizations	Special Purpose Vehicle which sub-contracts with not-for-profit organizations
Procurement approach	Annual closed contracting (extensions)	Competitive tendering for 5-year contract
Funding model	Costs-met, fee-for-service	Outcomes-based contract with payment contingent on pre-agreed participant-level outcome measures

In Kirklees, the delivery arrangement does not involve any profit-seeking organizations. KBOP is constituted as a ‘Social Prime’ with a dual mandate to enable both social and financial returns. Although a black box OBC is in place, it is the social prime that is party to the outcomes contract and the SLOs are not themselves subject to back-ended outcomes payments.

## Method

In this study, we investigate how a contracting reform shapes frontline service delivery in Kirklees using a longitudinal multi-method research design. The research received approval from University of Oxford’s Research Ethics Committee. In addition to the analysis of the contracts on paper, our data is structured across two organizational layers (as per Figure 1) with data for SLWs and SLMs (described below).

### Survey data and analysis: street-level workers

We use a 2-wave survey to gather standardized responses from the SLWs who undergo a transition from a fee-for-service arrangement to the impact bond contracting reform. We invited responses from all SLWs involved in service delivery in the fee-for-service contracts in early 2019 (Wave 1, *n* = 48). The second wave was conducted in 2021, once the KBOP impact bond was embedded (W2, *n* = 39).^3^

To explore the patterning of SLW perspectives and time use, the survey covers several characteristics of the interactions between SLWs and service users, as well as SLW’s perceptions of their relationship with their own SLMs (and organizations overall). The design of the survey was heavily informed by previous longitudinal studies that have tracked the shifting contractual arrangements in employment support systems (Considine 2001; Considine et al. 2015). We track indicators of personalization in client-servicing such as tailoring, user choice, caseworker – client ratios, and interactions with flanking services at wave 1 and wave 2. Additionally, in wave 2, we introduce questions focused on perceptions of the role of the SPV and dedicated training known to have been introduced since 2019. These questions were developed and tested with key stakeholders to ensure comprehension and appropriate terminology for SLWs. We also introduce open-text responses concerning SLW perceptions of personalization and reflections on the implications of the impact bond on their interaction with clients.

Administering two waves of the survey within the same delivery network allows us to explore shifts in SLW’s perceptions. Informed by the work of Considine et al. (2015), we analyse blocks of survey questions (shown in Appendix) to signal alignment to alternative service delivery norms and personalization practices. A key limitation is that these are self-report data and there has been some turnover of staff. However, we can see that there are no significant shifts in demographic details over time, and key features such as education level have not changed in a significant way between waves.

Guidelines recommend non-parametric tests for ordinal data, such as Chi-square (Pett 2015). Despite this, Likert-type categories are often treated as interval-level measurements without clear author explanations (Blaikie 2003). This study compares results using both t-tests (parametric) and chi-square (non-parametric).^4^ Table 2 outlines descriptive statistics for socioeconomic features combined for waves 1 and 2.

**Table 2.** Frontline survey descriptive statistics.

Variable/Survey Question	Obs	Mean	Std. Dev.	Min	Max
Female (binary)	71	.718	.453	0	1
White (binary)	87	.506	.503	0	1
College degree (binary)	87	.471	.502	0	1
Caseload (number of participants currently supporting)	82	15.829	6.707	3	31
Time spent with direct contact with participants (percentage)	77	49.857	17.073	10	100
How long do you tend to work with participants? (months)	79	12.494	2.669	3	14

When respondents are asked about gender, ethnicity and highest qualification attained several options are available, but here we present the results as binary for conciseness (considering the label as the most common answers). Respondents are also asked to determine what is the percentage of their average week performing several pre-determined activities. Here, we present only the disclosed ‘percentage spent with direct contact with participants’ for conciseness.

### Interview data and analysis: SLMs and key KBOP stakeholders

We conducted semi-structured interviews with SLMs operating in the legacy fee-for-service contract (10 interviewees in summer 2019) and following the implementation of the impact bond (19 interviewees in autumn 2021 – winter 22, during mid-implementation of the impact bond).^5^ Five SLMs were included in both research waves. Interviewees held different managerial roles and positions, ranging from team service manager to regional director. Semi-structured interviews were also conducted with the council contract managers (in 2019 and 2021, 5 in total), investment fund managers (5), KBOP board’s chair (1) and representatives from the managing social prime entity (KBOP, 10). Since the KBOP SPV was only introduced following the shift to an outcomes-contract, these interviewees only feature from 2021 onwards. Participants were selected using purposive sampling to ensure the involvement of experts from across the service array. Alongside this, snowball sampling was used to ensure representation from different types of organizations.

We use a semi-structured approach, and the topic guide is informed by previous analysis of social impact bonds (Carter et al. 2018). Interviews begin by asking managers about accountability and organizational imperatives, before investigating key contract terms and performance management across the contemporary contract. We explore the development of SLO-SLO interactions to assess connectivity to auxiliary flanking services. Finally, we ask questions about the level of service specification, co-production and person-centred delivery at the frontline.

We follow a two-phase coding approach using ATLAS-ti software. Firstly, structural coding applies a content-based or conceptual phrase to a segment of text data, relating to the research question (Saldaña 2021). We code the contracting era to which the text relates (e.g. later interviews would include both retrospective assessment of fee-for-service arrangements as well as contemporary statements on the evolving impact bond). Secondly, we apply descriptive coding to describe the topic of the data in a word or short phrase (Saldaña 2021). These descriptive codes identify facets of management and frontline delivery (e.g. standardization; person-led) to create a granular index of delivery practice. These sub-codes were subsequently bundled across the four governance dimensions identified in previous literature: procedural governance with control through rules, reliability and focus on universal treatment; corporate governance with emphasis on goal-driven plans and target-orientation; market governance with control through contracts and delivery focus on prices (or in the case of OBC, payable outcomes) and network governance with rationality of relationships, co-production and brokerage (Carter 2019; Considine 2001).

## Findings

### Street-level worker survey findings across fee-for-service and impact bond arrangements

Despite the introduction of a high-powered OBC and (as we shall see in interview data) intensive performance management function in the form of KBOP, the survey results find no significant change on key indicators of market orientation. At wave 2, under the impact bond arrangement, we expected SLWs to reveal a heightened attention to prices, competition and to the achievement of payable outcome measures. A key feature of our survey findings is the *absence* of change between wave 1 and wave 2 in dimensions associated with new public management, a target-led or competitive practice amongst SLWs (appendix).

Surprisingly, given the contractual imperative, the distribution of SLW responses does not substantively shift between waves in answer to: ‘The need to get an outcome is influential in determining what activities are included in the support I provide’. Similarly, we see no significant change in the degree to which SLWs acknowledge that they ‘do not like my competitors to know too much about how I get results’. One challenge in interpreting our survey findings is that under the impact bond SLW’s are potentially more sensitive in identifying and evidencing ‘outcomes’ and are more self-conscious in describing their practice as outcomes-oriented.

We do find one significant difference in a key indicator of substantive personalization described by SLWs themselves. The number of SLWs confirming that service users exert influence in shaping their individual support significantly increased (‘Participant’s activity preferences are influential in determining what activities are included in the support I provide’, *p* < 0.001; see Appendix). Somewhat strangely, this shift in user-centredness and tailoring is not accompanied by shifts in related survey items. There is no significant change in response to the question ‘If I want to, I can vary the service I provide to any service participant’. We discuss this below, as the formalization of a strengths-based approach under KBOP.

In terms of reported access to flanking services, there is little evidence of change amongst SLWs. Notably, the survey frames these questions in relation to *external* agencies. Under the outcomes-arrangement, SLWs are no more likely to acknowledge ‘When you get good results with a service participant it’s usually a team effort’.

**Figure 2.**
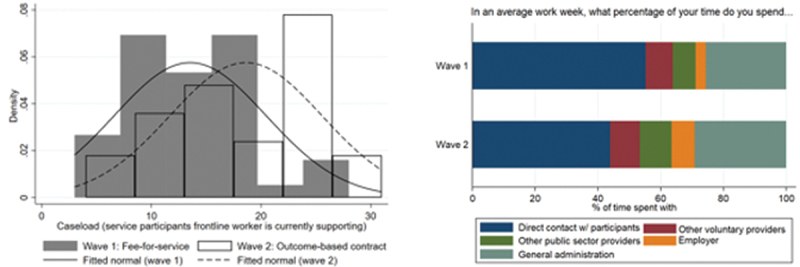
Caseloads and time use amongst street-level workers in fee-for-service (Wave 1) and social impact bond (Wave 2) contracting arrangements.

Figure 2 presents findings regarding caseload management. There is a substantive and statistically significant increase in caseload for SLWs under the impact bond arrangement (left-hand pane, Figure 2). Caseload per SLW increased from averages of 13 to 19 clients (an increase of 38%), where the difference in means is statistically significant. Beyond this, as outlined in the right-hand pane of Figure 2, there is a significant reduction in the proportion of SLW working time spent directly with service participants. Time with clients reduces from 55.2% in the legacy fee-for-service arrangement to 43.8% in the OBC (*p* < 0.01 considering simple t-tests). At the expense of direct time with participants, frontline staff spent more time working on every other mentioned activity (particularly ‘employer’ interactions and ‘general administration’). An additional question captures how long the worker usually supports each client, and there is no difference between waves (around 1 year for both).

### Findings from interview data under the fee-for-service contracting arrangement

SLMs emphasized the rule-bound auditing approach adopted by the council during the fee-for-service contracts, resulting in a process-driven management approach amongst SLOs. SLWs were encouraged to closely document activities and process targets were common. SLMs monitored and closely managed SLWs to ensure that they had completed and reviewed support plans and that risk reviews were conducted within specific timescales. Face-to-face visits and staff absences were also closely tracked and used as informal indicators of service functioning. Notably, there was no systematic recording of service outcomes for participants. A service manager explained

Because there is no formal recording of outcomes that’s not necessarily the thing that has been monitored. It’s more about ‘are you working with the customer? … Are you following the process?’ (Service manager, research wave 1)

To assess the quality of the service council staff used an auditing tool known as a ‘Quality Assessment Framework’ (QAF). Council staff would check – through annual validation visits – whether required policies were applied and whether quality client support was evidenced. SLMs experienced the QAF as very prescriptive and largely paper-based, creating a substantial administrative burden for providers to evidence contractual compliance. An SLO director described the process:

Under the Supporting People grant [fee-for-service], it was very, very prescriptive. So, we had to have a support plan that looked like this [gesture]. We had to record everything in the same way. We had to keep paper-based files that had to have this sheet and this sheet, and this sheet and it had to be ordered in this way’. (Provider director, research wave 2)

Another SLM explained

To comply with the QAF our organisation, as many others as well, built up these huge packs of policies that tell you how to do your job. (Services director, research wave 2)

Moreover, the process-driven nature of the QAF was associated with a particular form of ‘gaming’, i.e. providers putting in paper-based measures to give the image of a good service. Such practice – or fear of such practice – reduced the informative value of the QAF. This procedure-heavy and rule-bound approach accords strongly with constrained practice for personalization.

The fee-for-service contract featured three key performance indicators (KPI): service utilization, throughput, and ‘independent living’ and these are described as cutting against personalization practices. In particular, the utilization KPI was seen as influential and served to ensure that the service was used up to capacity. Interview findings suggest that ‘success’ was understood through the number of service participants on SLO registers. A senior service manager explained

‘It’s very specific about the number of people you should be supporting. … in order to have a utilisation figure that’s going to make their benchmark, you need to have that number of people in your service quite consistently’. (Senior service manager, research wave 1)

In the frontline practice of SLWs under the fee-for-service arrangement, we see descriptions of both standardized client interactions and target-oriented delivery. Service specifications prescribed service intensity: *‘you can only offer an hour a week’* (service manager, research wave 1). and managers did not give SLWs instructions to adapt support according to user needs. This made it challenging to address users’ underlying, complex needs and, hence, contributed to a ‘revolving door’ issue, i.e. clients re-entering support shortly after completing an intervention.

In 2019, frontline staff prioritized specific case load numbers whilst neglecting longer-term outcomes and personalization. A senior service manager reflected on issues in the legacy contract:

You could end up working in a way that isn’t necessarily very customer focused. Because it’s not about the outcomes necessarily, it’s not about the support you are providing. It’s just about the how many you’ve got on your service and how long they are on your service for. It can become a bit about numbers, rather than the actual quality. (Senior service manager, research wave 1)

### Findings from interview data under the impact bond contracting arrangement

The introduction of the impact bond arrangement brought changes for SLMs both in terms of their relationship with other SLOs and the emphasis of managerial support to SLWs. SLMs describe the emergence of greater awareness of fellow SLO’s performance. This focus on ‘competitor’s’ performance was induced by the contract’s emphasis on payable outcomes and delivery group meetings in which KBOP SPV management compared SLO performance. A head of services explained:

… you’re very aware of everybody else’s performance. Performance is something that’s presented to you in the meetings, so you’re very concerned about your performance against that of other providers and I think that that can have a negative effect on the collaboration between providers. I don’t see it as a motivator. We are already motivated to do well. (Head of services, research wave 2)

Evidence on the coherence between the indicators of personalization practice across SLW and SLM level is mixed. On the one hand, open-text survey responses from SLWs describe the need to achieve outcomes as the imperative for support:

I struggle sometimes to achieve the targets set as the asset strength-based model should be needs-led and achieved in the client’s time, however there is a sense that meeting the outcomes is a priority and I do feel under pressure at times to meet these targets, there is a real conflict between these areas (SLW, survey wave 2).

Senior managers, particularly those at a director level, were more at ease with the congruence between a strengths-based approach and the achievement of measurable outcomes. SLMs indicated that the outcomes-based and personalized approaches are synergistic, i.e. person-led support naturally leads to outcome achievements. As the director of an SLO (wave 2) explained

We are still very outcomes-focused in terms of the managers and monitoring the outcomes; and the support workers still having to evidence that they’ve got the outcomes. But in the support sessions, there isn’t the same focus on the outcomes as there used to be. The focus is on the relationship with the person. And then the outcomes come anyway. In fact, they’ve increased.

Findings from interviews with SLMs suggest the enactment of network governance in service management. This relates to a focus on personalized ‘asset-based’ support, the use of flexible management tools, granting autonomy to frontline staff, and strong collaboration between network stakeholders.

The SPV management team proactively promoted frontline discretion by applying a more developed leadership approach. The director of the investment fund depicts this:

But the main goal in the first year is more psychological - it’s getting the right culture and the right approach. Because if this is all about trying a set of experiments and delivery pilots, you need those pilots to be bubbling up from the front line, from everybody. And so you need everybody to appreciate that they have the opportunity to contribute to improving the service and redesigning it. And everyone should be sort of coming up with ideas for what to do differently. (Investment fund director, research wave 2)

Deeper procedural and substantive personalization may be enabled by SLMs granting SLWs discretion in defining frequency, length and substance of support. A service director explained

It does enable you to change your mode of service delivery because the only constant you need is the paid outcomes. (Service director, research wave 2)

Managers describe horizontal forms of professional accountability and enhanced collaboration between delivery partners in the OBC arrangement, aligning with network governance. Important features include both ‘co-governance’ since providers participate in the design and planning of the service and ‘co-management’ for decisions in resourcing and delivery.

KBOP’s new collaborative infrastructure enabled active participation in the planning and design of services via dedicated forums for operational service development and the promotion of service personalization, facilitated by the SPV management. The shared outcomes framework fostered a sense of shared accountability, as SLO managers recognized that succeeding in the outcomes contract requires a joint effort. Comparing provider collaboration across the legacy fee-for-service and OBC arrangements, a senior operations manager noted

I can see that we are working more consistently as a group of providers, [which] I think is a benefit. Because it helps with benchmarking and an expectation around what we’re delivering. And that helps with a consistency of the service and the level of service and the quality that we might expect. Whereas I don’t think that there was any mechanism for that with the group of contracts previously. (Senior operations manager, research wave 2)

The SPV management team supported resource-pooling and information-sharing that unlocked access to previously unavailable flanking services, such as mental health counselling. The same senior operations manager explained

like maybe we can access some of their other services, or we can tap into some of the training that they may deliver, or, you know, those are the kind of added benefits that those partnership working arrangements should bring. (Senior operations manager, research wave 2)

## Discussion

There is some dissonance in our findings; in this section, we consider evidence for changes in procedural and substantive personalization, taking these concepts in turn. An important discontinuity is the reform to referral assessment and support plans. Prior to the OBC, SLOs each managed their own ad-hoc referrals. This led to competition for clients and long waiting lists. Under the OBC, referrals are coordinated by a central KBOP hub. Initial motivational interviews function as assessments and referrals are then allocated according to providers’ expertise, capacity, and the complexity of client needs. In terms of the referral *process*, we see standardization and a reduction in SLW discretion via the single service ‘front door’. But in terms of language used and the referral *approach*, there is a marked shift towards a more person-centred model. Clients are no longer expected to recount their story multiple times, and the assessment conversation has shifted away from a deficit model. This reveals limitations in the survey instrument which cannot get beneath the surface to explore different elements of SLW–client interaction to unpack dualisms of procedural ‘standardising’ (referral routes and hand-over points between agencies) whilst ‘personalising’ (in language and interactions at the frontline).

There is strong evidence for procedural personalization as the frequency and form of support sessions is more varied. Under the OBC arrangement, interactions are guided by participant preferences:

The individual has the choice, power with, not power over. (SLW, open text survey response, research wave 2)

Predominantly, our findings suggest that substantive personalization is enhanced under the OBC through i) expansion and co-production of flanking services and ii) staff development and training (discussed below). However, increased caseload and heightened administrative burden, related to increased reporting demands, leave less time for participant support, and may present barriers to substantive personalization of the service.

Contrasting with previous research where OBC reforms are associated with an excessively lean and generic support offer (McGann 2023; O’Sullivan, McGann, and Considine 2021) in our study, the KBOP SLOs have been funded to hire additional support specialists. An Education-Training-Employment coordinator builds providers’ capacity to support meaningful employment trajectories, where previously no employment support was available. A mental health specialist, providing preventative clinical services, expands the service offer to accommodate varied needs, making ‘flanking’ services available.

A flexible and discretionary ‘personalisation fund’ enables KBOP SLWs – in consultation with clients – to co-commission items or services that augment the service offer. A degree of financialised discretion does apply at the frontline, but this is framed as discretion to spend an enhanced budget to secure outcomes. The KBOP management team and SLMs encourage SLWs to access and spend the personalization fund to better meet the aspirations and preferences of participants. Such financialised discretion is structured to expand tailoring and augment the service offer rather than to curtail the availability of provision as seen in parking practices of previous OBC initiatives like the Work Programme in the UK (Rees, Whitworth, and Carter 2014). The importance of the fund is underscored by a service manager:

There’s the personalisation fund … it’s just an absolute gift. We’ve been able to use that to access counselling for people who would have sat on a waiting list for years … There’s much more room within this to think outside the box and then to get practical help to do some of these things via the personalisation fund. It was very limited under the old contract. (Service manager, research wave 2)

Under the KBOP OBC, the scope and appropriateness of the service offer is actively shaped through elements of co-production (Bovaird 2007; Lindsay et al. 2018; Needham 2011a). KBOP hosts a monthly co-production forum, where people with lived experience co-design service improvements such as designing approaches to prevent client drop-out. People with lived experience also co-deliver as peer mentors.

Where previous research indicates that OBC reforms precipitate de-skilling and excessive routinization in case management work (McGann 2023) our study instead identifies important training elements and a galvanizing professional identity for SLW around ‘asset-based’ practice. Under KBOP, SLWs received training in strengths-based service provision to ensure that each has the skillset to advance person-centred delivery. Training in active listening and coaching underpins a tailored, integrated service model that gives people choice and autonomy over their own support (Baraki and Phagoora 2022). Emphatic endorsement of the ‘strength-based’ approach from KBOP leadership team perhaps explains why SLWs report that participant’s preferences are influential in shaping support whilst we see no shift for SLWs’ answer to ‘If I want to I can vary the service I provide to any service participant’. Under KBOP leadership, SLWs should not deviate from the strength-based approach.

The policy implication is that blunt application of OBC will not *automatically* unlock promises of personalization and effectiveness. OBC is not a singular instrument and in a context of heavy price competition, unrealistic performance expectations, and heavily back-ended payment models an opaque black box can enable provider profiteering at the expense of service quality, equity and substantive personalization. The level of resourcing inside an OBC arrangement, particularly the availability and emphasis of working capital may play an important moderating role, shaping frontline practice. Public managers should beware of the simplistic ‘black box’ and consider other forms of accountability tools that will enable them both to understand and assure services are meeting expectations of quality and person-centredness. A pragmatic response could be the adoption of a ‘grey box’ to uphold key aspects, such as sufficiently low case-loads (a precondition of substantive personalization (van Berkel and Knies 2016)) and/or a ‘formal-relational’ set of norms and expectations. To overcome concerns in marketized arrangements public managers will need to focus on *long-term and purposeful* partnership models rather than short ‘transactional’ contracts and consider investment in workforce development to enable more effective forms of contract management with SLOs.

## Concluding remarks

We find that flexible, outcomes-based contracting, through an impact bond with devolved responsibility to SLOs, enables more personalized and responsive services compared to more conventional fee-for-service contracting arrangements. It is important to acknowledge that as with broad ‘marketisation’ reforms, OBC is not a singular phenomenon but rather captures a diverse range of ‘practices, rationales, trajectories, actors and impacts’ (Meagher and Goodwin 2015, 4). In response to our research question, we find that the introduction of an impact bond arrangement enables managerial practice that connects across SLO boundaries in a form of network accountability and connects to flanking services. SLWs describe being more attuned to co-production via an asset-based approach, with greater attention being placed on clients shaping their own support. Curiously, we find no increase in SLWs reporting that their work is responsive to prices or to competition under the impact bond, but we do detect an increase in caseloads, which may hinder deeper forms of substantive personalization.

This study suffers from three key limitations. First, the findings are not readily generalizable to other OBCs, and specificities of the UK context and the involvement of impact investors should be considered in drawing inferences from our findings. Second, research participants might have a vested interest in seeing the partnership continue, which may encourage a positive response bias, particularly across service managers. Although we have tried to circumvent this by incorporating interviews with additional stakeholders such as council managers and by conducting documentary analysis, such issues are not entirely resolved. Third, in the frontline staff survey, we face the common problems related to small-N analysis. Survey responses relied on self-reported data; in-depth exploration of frontline behaviours and attitudes through observations and interviews need to be addressed in future research.

Financialised discretion in contractualised welfare services needs to be considered as a doubled-faced construct. Such ‘black box’ discretion in OBC arrangements *can* be associated with practices that corrode public value, with low-cost, poor-quality provision short-circuiting to provider profits (Fuertes and Lindsay 2016; Wiggan 2015). Here, we add to the literature by identifying an *enabling* form of financialised discretion where contractual flexibilities, in a (reasonably) well-resourced, outcomes-oriented environment, with commitment to long-term partnership can bring about forms of tailoring and asset-based working that accord with greater personalization. In this alternative context of an impact bond, we see that financialised discretion may – at least partially – augment both procedural and substantive personalization.

Those who want to meaningfully expand the availability of person-centred, asset-based support need to consider not just contractual incentives but consider what the outcomes of a quality service should cost (to avoid excessive price discounting and unrealistic performance expectations); working capital requirements for SLOs; and assurances around a shared, long-term focus on meaningful outcomes for programme participants.

## Notes

1. This is a description of a ‘pure’ black box approach. In practice, there may be a spectrum ranging from full black box to fully specified service contracts and government commissioners may pursue a grey box (see Wiggan 2015). There are a range of approaches that public managers may use in an attempt to regulate service quality and participant experience within a black box.

2. The service was designed so that the KBOP SPV functions as a node in the referral process that enables potential participants to access support. Before the outcomes contract was launched, Kirklees Council was responsible for procuring and implementing a digital referral and case management system.

3. The response rate is 46% for W2. We can’t provide a precise response rate for W1 since, during this first wave of survey administration, the research team were reliant on SLMs to distribute links to the survey across SLWs in their teams. Given that N is larger at W1 and the service itself has not reduced in size, we expect that the response rate at W1 > 46%.

4. Regression-based estimates are also available upon request. Findings are always maintained.

5. Interviews in the first research wave were conducted face-to-face. In the second research wave, following COVID-19, most of the interviews were conducted remotely using MS Teams.
